# The enzootic life-cycle of *Borrelia burgdorferi* (*sensu lato*) and tick-borne rickettsiae: an epidemiological study on wild-living small mammals and their ticks from Saxony, Germany

**DOI:** 10.1186/s13071-017-2053-4

**Published:** 2017-03-13

**Authors:** Anna Obiegala, Nina Król, Carolin Oltersdorf, Julian Nader, Martin Pfeffer

**Affiliations:** 1 Institute of Animal Hygiene and Veterinary Public Health, University of Leipzig, An den Tierkliniken 1, 04103 Leipzig, Germany; 2 Department of Microbial Ecology and Environmental Protection, Institute of Genetics and Microbiology, University of Wrocław, Przybyszewskiego 63/77, 51-148, Wrocław, Poland

**Keywords:** *Ixodes ricinus*, *Dermacentor reticulatus*, Borrelia MLST, *Myodes glareolus*

## Abstract

**Background:**

*Borrelia burgdorferi* (*sensu lato*) and rickettsiae of the spotted fever group are zoonotic tick-borne pathogens. While small mammals are confirmed reservoirs for certain *Borrelia* spp., little is known about the reservoirs for tick-borne rickettsiae. Between 2012 and 2014, ticks were collected from the vegetation and small mammals which were trapped in Saxony, Germany. DNA extracted from ticks and the small mammals’ skin was analyzed for the presence of *Rickettsia* spp. and *B. burgdorferi* (*s.l*.) by qPCR targeting the *gltA* and *p41* genes, respectively. Partial sequencing of the rickettsial *ompB* gene and an MLST of *B. burgdorferi* (*s.l*.) were conducted for species determination.

**Results:**

In total, 673 small mammals belonging to eight species (*Apodemus agrarius*, *n* = 7; *A. flavicollis*, *n* = 214; *Microtus arvalis*, *n* = 8; *Microtus agrestis*, *n* = 1; *Mustela nivalis*, *n* = 2; *Myodes glareolus*, *n* = 435; *Sorex araneus*, *n* = 5; and *Talpa europaea*, *n* = 1) were collected and examined. In total, 916 questing ticks belonging to three species (*Ixodes ricinus*, *n* = 741; *Dermacentor reticulatus*, *n* = 174; and *I. trianguliceps*, *n* = 1) were collected. Of these, 474 ticks were further investigated. The prevalence for *Rickettsia* spp. and *B. burgdorferi* (*s.l*.) in the investigated small mammals was 25.3 and 31.2%, respectively. The chance of encountering *Rickettsia* spp. in *M. glareolus* was seven times higher for specimens infested with *D. reticulatus* than for those which were free of *D. reticulatus* (OR: 7.0; 95% CI: 3.3–14.7; *P* < 0.001). In total, 11.4% of questing *I. ricinus* and 70.5% of *D. reticulatus* were positive for *Rickettsia* spp. DNA of *B. burgdorferi* (*s.l*.) was detected only in *I. ricinus* (5.5%). Sequence analysis revealed 9 *R. helvetica*, 5 *R. raoultii*, and 1 *R. felis* obtained from 15 small mammal samples.

**Conclusion:**

Small mammals may serve as reservoirs for *Rickettsia* spp. and *B. burgdorferi* (*s.l*.). While the prevalence for *Rickettsia* spp. in *M. glareolus* is most likely depending on the abundance of attached *D. reticulatus*, the prevalence for *B. burgdorferi* (*s.l*.) in small mammals is independent of tick abundance. *Dermacentor reticulatus* may be the main vector of certain *Rickettsia* spp. but not for *Borrelia* spp.

**Electronic supplementary material:**

The online version of this article (doi:10.1186/s13071-017-2053-4) contains supplementary material, which is available to authorized users.

## Background

Tick-borne diseases require invertebrate vectors (ticks) and vertebrate hosts for the completion of their life-cycle [1, 2]. Two of the most common tick species in Europe - and at the same time the most important vectors - are the castor bean tick *Ixodes ricinus* and the meadow tick *Dermacentor reticulatus*. Their immature life stages (larvae and nymphs) parasitize mostly on small-sized birds and on small mammals. This is why small mammals are essential for the maintenance and distribution of ticks and thus tick-borne diseases [3–7].

*Borrelia burgdorferi* (*sensu lato*) is the causative agent of Lyme disease (LD) which is considered the most common tick-borne disease in Europe and North America [8, 9]. *Borrelia burgdorferi* (*s.l*.) is a complex of gram-negative bacteria belonging to at least 20 genospecies from which nine occur in Europe [10]: *B. afzelii*, *B. bavariensis*, *B. bissetti*, *B. burgdorferi* (*sensu stricto*)*, B. finlandensis*, *B. garinii*, *B. lusitaniae*, *B. spielmanii* and *B. valaisiana. Borrelia burgdorferi* (*s.l*.) is mainly transmitted by *I. ricinus* ticks in which transovarial transmission was recorded for *B. miyamotoi* but not for genospecies belonging to the *B. burgdorferi* (*s.l*.) complex [11]. Over 40 vertebrate species, in particular small mammals, are considered reservoir hosts for *B. burgdorferi* (*s.l*.) [12, 13].

*Rickettsia* spp. are divided into four groups: the spotted fever group (SFG), the typhus group, the ancestral group and the transitional group [14, 15]. Tick-borne rickettsioses are caused by obligate intracellular gram-negative bacteria from the SFG. *Ixodes ricinus*, *D. reticulatus* and *Rhipicephalus* spp. are mainly involved in the circulation of pathogenic *Rickettsia* species in Europe (such as *R. aeschlimannii*, *R. conorii*, *R. helvetica*, *R. massiliae*, *R. monacensis*, *R. raoultii*, *R. sibirica* and *R. slovaca*). Transovarial and transstadial transmission has been observed in these tick species. DEBONEL (*Dermacentor*-borne necrosis erythema lymphadenopathy) also known as TIBOLA (tick-borne lymphadenopathy) syndrome is transmitted by *D. reticulatus* and associated with *R. slovaca* and *R. raoultii* [15–19]. Wild boars (*Sus scrofa*) and domestic ruminants are considered as potential reservoirs for *R. slovaca.* Additionally, sika deer (*Cervus nippon*), dogs (*Canis lupus familiaris*), common rabbits (*Oryctolagus cuniculus*) and lizards (*Teira dugesii*) are potential reservoirs for *R. helvetica*, *R. conorii*, *R. massiliae* and *R. monacensis,* respectively [15, 17, 20–24]. However, the reservoir of *R. raoultii* is still not established.

Prevalence rates for *Borrelia* spp. and *Rickettsia* spp. in *I. ricinus* ticks in Germany differ and can reach levels of 34 and 61%, respectively [25–31]. In Germany, the investigations of *Rickettsia* spp. in wild-living small mammals are scarce and were conducted mostly on *Myodes glareolus*, *Apodemus flavicollis* and *Erinaceus europaeus* [32–34]. Earlier, *Borrelia* spp. was detected in small animals such as *Glis glis*, *E. europaeus*, *A. flavicollis* and *Mus musculus* in Germany [35–37]. However, all studies previously published on *Borrelia* spp. in small mammals from Germany were focused on the detection of a single locus (*ospA* gene). In the present study, multi-locus sequence typing (MLST) of eight housekeeping genes was conducted in order to detect different sequence types of *B. burgdorferi* (*s.l*.) in small mammals.

The aims of this study were (i) detection of tick-borne rickettsiae and *B. burgdorferi* (*s.l*.) by qPCR in captured small mammals and in the questing ticks from selected suburban areas in Saxony, Germany; (ii) species identification of these pathogens by conventional PCR and MLST; and (iii) comparison of prevalence rates of *B. burgdorferi* (*s.l*.) and of tick-borne rickettsiae between the respective small mammals and tick species.

## Methods

### Study sites

From 2012 to 2014, small mammals as well as questing ticks were collected at six different study sites in and near the city of Leipzig in Saxony, Germany. Previously, these study sites were described in detail and consecutively named from “E” to “I” (E: 51°16'27.6"N, 12°19'18.8"E; F: 51°17'13.0"N, 12°20'40.2"E; G: 51°16'20.3"N, 12°23'12.7"E, H1: 51°18'14.6"N, 12°24'41.4"E; H2: 51°17'35.5"N, 12°24'07.5"E, I: 51°18'01.2"N, 12°22'09.5"E) by our group [38]. Three of those six study locations (sites E, F and G) surround a lake which was artificially created from a former brown coal mining area and which is now often frequented by visitors for recreational activities. Site “H” is subdivided in two small areas located in a recreational city park which was created from a former waste disposal area. Site “I” is a part of one of the largest riparian forests in Middle Europe and is located near the city centre of Leipzig. Sites “I” as well as “G” were only investigated in 2012 due to financial restrictions (see complete sequence batches in Additional files 1 and 2).

### Small mammals and their attached ticks

Small mammals were captured from March to October in 2012, from January to November in 2013, and from January to October in 2014. Each month, twenty Sherman© live animal traps (H. B. Sherman Traps, Inc., Tallahassee, Fla., USA) were baited with apple slices and placed at each study site for two consecutive nights. Captured small mammals were immediately anesthetized with CO_2_ and subsequently euthanized by cervical dislocation (local permit numbers: 36.11-36.45.12/4/10-026-MH, 364.60/2009-102-2). By the use of taxonomic keys, captured animals were morphologically identified [39]. For the present study, the ectoparasites (ticks in particular) were additionally collected from their bodies. Skin samples as well as ticks, which were morphologically identified [40] in advance, were stored at -80 °C until further processing.

### Collection of questing ticks

Simultaneously to each rodent trapping action, questing ticks were collected monthly by the use of the flagging method at each study site. The ticks were morphologically identified and stored individually at -80 °C until further processing [40].

### Tissue preparation and DNA extraction

Skin samples were taken individually and then 0.6 g of sterile steel beads (sized 2.8 mm, Peqlab Biotechnologie, Erlangen, Germany) as well as 600 μl phosphate buffered saline were added to each sample. Moreover, 0.6 g ceramic beads (sized 1.4 mm, Peqlab Biotechnologie) and 200 μl PBS were added to each engorged or questing tick. All samples were homogenized at 5700× *rpm* for 20 s in the Precellys®24 tissue homogenizer (Bertin Technologies). Subsequently, DNA was extracted from all samples with the QIAamp DNA Mini Kit (Qiagen, Hilden, Germany) according to the manufacturer’s recommendations for tissue DNA extraction. The quality and the quantity of the DNA samples were measured with a spectrophotometer (NanoDrop® 2000c, Peqlab Biotechnologie).

### PCR methods

Initially, small mammal and tick DNA samples were screened for the presence of *Rickettsia* spp. and *Borrelia burgdorferi* (*s.l*.) by qPCR. Real-time PCR analysis targeting the citrate synthase gene (*gltA*, 70 bp) was performed for *Rickettsia* spp. as previously described [41]. The initial screening for *Borrelia burgdorferi* (*s.l*.) which is targeting the *p41* flagellin gene (96 bp) was carried out following a previously published protocol [42].

All *Rickettsia*-positive samples yielding a cycle threshold value (CT) below 35 were further analysed by a conventional PCR targeting 811 bp of the outer membrane protein B gene (*ompB*) of SFG rickettsiae [43]. A 1.5% agarose gel was stained with Midori Green (NIPPON Genetics, Düren, Germany) and PCR products were analysed under UV illumination. Five randomly selected samples which were positive for *B. burgdorferi* (*s.l*.) by real-time PCR and yielded a CT value below 33 were further analysed by multi-locus sequence typing (MLST) targeting the following housekeeping genes: *nifS*, *pyrG*, *clpX*, *pepX*, *uvrA*, *rplB*, *cplA* and *recG* [44]. For all genes a semi-nested or a nested approach was performed as described, however with slight modifications. The first amplification step for the genes *clpX, rplB, pepX* as well as the second amplification step for the genes *rplB, clpA* and *clpX* were performed with a touchdown protocol with 11 cycles with annealing temperatures ranging down from 56 to 46 °C, and further 34 cycles with an annealing temperature of 46 °C. The first amplification step of the *nifS* gene was likewise a touchdown protocol with nine cycles with annealing temperatures ranging down from 51 to 43 °C, and further 36 cycles with an annealing temperature of 46 °C. The annealing temperature of the *nifS* gene in the second amplification step was 51 °C as for the *uvrA* gene in both amplification steps. The annealing temperature for the first amplification step of the *recG* gene and for the second amplification step of the *pepX* gene was 55 °C. The annealing temperature of the first amplification step of the *pyrG* gene and the *clpA* gene was 47 °C. The annealing temperature in the second amplification step was 49 °C for the *pyrG* gene and 50 °C for the *recG* gene.

Sequencing was performed commercially (Interdisziplinäres Zentrum für Klinische Forschung, Leipzig, Germany) for both, *Rickettsia* spp. and *Borrelia* spp. MLST, with forward and reverse primers of each gene used for PCR amplification. Results were analysed with the Bionumerics Software (Version 7.6.1. Applied Maths, Inc., Austin, TX USA). Sequences were aligned to available data in GenBank with BLASTn (http://blast.ncbi.nlm.nih.gov/Blast.cgi) Obtained MLST sequences were aligned and compared to sequences from the MLST database (http://pubmlst.org/borrelia).

### Statistical analysis

Confidence intervals (95% CI) were determined for prevalences of *Rickettsia* spp. and *B. burgdorferi* (*s.l*.) in small mammals and in the questing ticks by the Clopper and Pearson method with the use of the Graph Pad Software (Graph Pad Software Inc., San Diego, Ca., USA). Pearson’s Chi-squared test was used with a type I error α of 0.05 to test the independence of compared prevalences. Fisher’s exact test was used for small sample sizes (*n* < 30) (Graph Pad Software). The odds ratio was calculated testing the association between the *D. reticulatus* ticks burden on *Myodes glareolus* and the prevalence of *Rickettsia* spp. in *M. glareolus*.

## Results

### Collection of small mammal samples

Altogether, 673 small mammals belonging to eight species (*Apodemus agrarius*, *n* = 7; *A. flavicollis*, *n* = 214; *Microtus arvalis*, *n* = 8; *Microtus agrestis*, *n* = 1; *Mustela nivalis*, *n* = 2; *Myodes glareolus*, *n* = 435; *Sorex araneus*, *n* = 5; *Talpa europaea*, *n* = 1) were collected. In 2012, a total of 454 small mammals were trapped: 306 *M. glareolus*; 127 *A. flavicollis*; 8 *Mi. arvalis*; 4 *A. agrarius*; 5 *S. araneus*; 2 *Mu. nivalis*; 1 *Mi. agrestis*; and 1 *T. europaea*. In 2013, only 90 small mammals were captured: 42 *M. glareolus* and 48 *A. flavicollis.* In 2014, a total of 129 small mammals were captured: 87 *M. glareolus*, 39 *A. flavicollis* and 3 *A. agrarius.*

### Tick infestation on small mammals

Overall 3330 ticks were collected from 602 small mammals in the years 2012 (*n* = 1728), 2013 (*n* = 475) and 2014 (*n* = 1127). All small mammal species were infested with ticks except for *Sorex araneu*s, *Microtus agrestis* and *Talpa europaea*. Totals of 310 *D. reticulatus* (159 larvae and 151 nymphs), 2802 *I. ricinus* (2583 larvae and 219 nymphs), 3 *I. trianguliceps* (3 nymphs), 208 *Ixodes* spp. (187 larvae and 21 nymphs), and seven ticks which could not be identified due to damage, were collected. Data on tick infestation per small mammal species are shown in Table 1.

**Table 1:** Collected ticks from small mammals per tick species, developmental stage and per small mammal species

Tick species and developmental stage	No. of ticks collected	Number of ticks per small mammal species/number of small mammals infested				
		*Myodes glareolus*	*Apodemus flavicollis*	*Apodemus agrarius*	*Mustela nivalis*	*Microtus arvalis*
*Ixodes ricinus*	2802	1439/391	1281/186	65/7	1/1	16/4
Larva	2583	1290/385	1219/179	59/7	1/1	14/4
Nymph	219	149/87	62/22	6/3	–	2/2
*Ixodes trianguliceps*	3	3/3	–	–	–	–
Nymph	3	3/3	–	–	–	–
*Ixodes* spp*.*	208	111/61	87/32	9/2	–	1/1
Larva	187	100/56	81/30	5/2	–	1/1
Nymph	21	11/9	6/2	4/1	–	–
*Dermacentor reticulatus*	310	293/32	3/3	14/2	–	–
Larva	159	151/29	3/3	5/1	–	–
Nymph	151	142/25	–	9/2	–	–
Tick^a^	7	6/6	1/1	–	–	–

^a^Not identified (damaged)

### Collection of questing ticks

Altogether 916 questing ticks were collected: 741 *I. ricinus* (79 females, 105 males, 504 nymphs and 53 larvae), 174 *D. reticulatus* (72 females and 102 males) and one *I. trianguliceps* (female). The breakdown of ticks by year and life-cycle stage is shown in Table 2.

**Table 2:** Prevalence of *Borrelia burgdorferi* (*s.l*.) and of *Rickettsia* spp. in ticks from 2012 to 2014 in Saxony, Germany

Tick species and developmental stage	No. of ticks collected	No. of ticks selected for further study	No. of ticks positive for *Rickettsia* spp. by qPCR (%)	No. of ticks positive for *Borrelia* spp. by qPCR (%)
*Ixodes ricinus* (Total)	741	366	42 (11.4)	20 (5.5)
Larvae	53	10	0 (0)	0 (0)
Nymphs	504	229	30 (13.1)	16 (6.9)
Adults	184	127	12 (9.5)	4 (3.1)
*Ixodes trianguliceps* ^a^	1	1	1 (100)	0 (0)
*Dermacentor reticulatus* ^a^	174	105	74 (70.5)	0 (0)
Total	916	472	117 (24.8)	20 (4.2)

^a^Adult ticks only

### PCR analysis for *Rickettsia* spp. and *Borrelia burgdorferi* (*s.l*.) in small mammals

In total, 210 out of 673 small mammals were positive for *Borrelia burgdorferi* (*s.l*.) (31.2%; 95% CI: 27.8–34.8). Of these, 140 out of 454 small mammals in 2012 (30.8%; 95% Cl: 26.9–35.5), 22 out of 90 (24.4%; 95% CI: 16.7–34.3) in 2013, and 48 out of 129 (36.7%; 95% Cl: 28.6–44.9) in 2014 were positive for *B. burgdorferi* (*s.l*.) detected by qPCR. Pairwise comparisons for the prevalence between the years revealed no significant differences. The prevalence in both dominant small mammal species was high, 32.9% (95% CI: 28.6–37.4) for *M. glareolus* and 25.4% (95% CI: 28.6–37.4) for *A. flavicollis*. Interestingly, these prevalence values did not differ significantly (*P* = 0.5302).

Due to financial restrictions, only five *M. glareolus* samples were tested by MLST. All sequenced samples were positive for *B. afzelii*. While four samples had the sequence type (ST) 165 (sample ID “321–324” in the *Borrelia burgdorferi* MLST database), one sample had the ST 559 (sample ID “1565”) (see complete sequence batches in Additional files 1 and 2).

Regarding the prevalence of *Rickettsia* spp., overall 170 of 673 small mammals (25.3%; 95% CI: 22.1–28.7) were positive. In 2012, a total of 134 out of 454 small mammals (29.7%; 95% CI: 22.1–28.7), in 2013, only 4 out of 90 (4.0%; 95% CI: 1.4–11.2) and in 2014, a total of 32 out of 129 small mammals (24.8%; 95% CI: 16.3–35.7) were positive for *Rickettsia* spp. detected by qPCR. The prevalence was significantly lower in 2013 compared to both of the other years (*P* < 0.0001). The chance of encountering *Rickettsia* spp. in *M. glareolus* was seven times higher for individuals infested with *D. reticulatus* than for specimens which were free of *D. reticulatus* (OR: 7.0; 95% CI: 3.3–14.7; *P* < 0.0001). In total, 17 samples (12 *M. glareolus* and 5 *A. flavicollis*) were sequenced. Altogether 15 records were available from these 17 samples. Two could not be further determined by sequencing. Nine samples were positive for *R. helvetica* (4 *A. flavicollis* and 5 *M. glareolus*), five for *R. raoultii* (all *M. glareolus*) and one for *R. felis* (*A. flavicollis*). All *R. raoultii*-positive *M. glareolus* were infested with *D. reticulatus* ticks. All *R. helvetica*-positive small mammals were infested with *I. ricinus* or had no tick at all, except for one *M. glareolus* which was simultaneously infested with *I. ricinus* and *D. reticulatus.* All sequences positive for *R. helvetica* showed 100% identity to a sequence in GenBank (KU310591) which was earlier obtained from an *I. persulcatus* tick from Russia (Katarshov et al. unpublished). All sequences positive for *R. raoultii* showed 100% identity to a sequence in GenBank (KU961542) which was earlier obtained from a *D. marginatus* tick from Russia (Katarshov et al. unpublished). The single *R. felis* sequence showed 100% identity to a sequence in GenBank (GU324467) which was also obtained from *A. flavicollis* in Germany [33]. The prevalence and the distribution of *Borrelia* spp. as well as *Rickettsia* spp. for all small mammal species are shown in Table 3.

**Table 3:** Prevalence of *Borrelia burgdorferi* (*s.l*.) and *Rickettsia* spp. in small mammals collected from 2012 to 2014 in Saxony, Germany

Small mammal species	No. of small mammals captured	No. of small mammals positive for *Rickettsia* spp. by qPCR (%)	No. of small mammals positive for *Borrelia* spp. by qPCR (%)	No. of samples selected for identification of *Borrelia* spp. (MLST)	No. of samples selected for identification of *Rickettsia* spp. (*ompB*)	Species identification for *Rickettsia* spp.
*Apodemus flavicollis*	214	50 (23.4)	64 (25.4)	0	5	4× *R. helvetica*; 1× *R. felis*
*Apodemus agrarius*	7	0 (0)	3 (42.9)	0	0	
*Myodes glareolus*	435	114	143 (32.9)	5 (4× ST 165; 1× ST 559)	12^a^	5× *R. raoultii*; 5× *R. helvetica*
*Microtus arvalis*	8	0 (0)	5 (62.5)	0	0	
Other^b^	9	0 (0)	0 (0)	0	0	
Total	673	170 (25.3)	210 (31.2)	5	12	9× *R. helvetica;* 1× *R. felis*; 5× *R. raoultii*

^a^Two samples could not be further determined, amplification was not possible by conventional with the target gene *ompB*

^b^Other: *Mustela nivalis* (*n* = 2); *Sorex araneus* (*n* = 5); *Talpa europaea* (*n* = 1); *Microtus agrestis* (*n* = 1)

### PCR analysis for *Rickettsia* spp. and *Borrelia burgdorferi* (*s.l*.) in questing ticks

Altogether 4.2% (95% CI: 2.7–6.5) of the examined questing ticks were positive for *Borrelia burgdorferi* (*s.l*.). All positive ticks were *I. ricinus* (5.5%; 95% CI: 3.5–8.3); none of the 105 *D. reticulatus* (95% CI: 0.0–2.8) examined nor the single *I. trianguliceps* were positive for *Borrelia burgdorferi* (*s.l*.). The prevalences did not differ significantly between the years 2012–2014 (*P* = 0.298). The prevalence was significantly higher in *I. ricinus* than in *D. reticulatus* (*P* = 0.01). Furthermore, the prevalence of *B. burgdorferi* (*s.l*.) was significantly higher in small mammals than in questing *I. ricinus* (*P* < 0.0001).

Overall, 24.8% of all examined ticks were positive for *Rickettsia* spp. (95% CI: 21.1–28.8). The prevalence in ticks did not differ significantly between the years (*P* = 0.288). The prevalence was 11.4% (95% CI: 8.6–15.2) in *I. ricinus* and 70.5% in *D. reticulatus* (95% CI: 61.1–78.4). The single *I. trianguliceps* was positive for *Rickettsia* spp. Regarding both dominant tick species collected, the prevalence was significantly higher in *D. reticulatus* than in *I. ricinus* (*χ* ^2^ = 164.42, *P* < 0.0001). Overall, the prevalence in ticks compared to the small mammals did not differ significantly (*χ* ^2^ = 0.013, *df* = 1, *P* = 0.889). However, the prevalence in *D. reticulatus* ticks was significantly higher than in small mammals (*χ* ^2^ = 84.18, *df* = 1, *P* < 0.0001).

## Discussion

This study was focussed on the detection of *Borrelia burgdorferi* (*s.l*.) and rickettsiae of the spotted fever group in wild-living small mammals and questing ticks from Germany. *Borrelia burgdorferi* (*s.l*.) is the causative agent of Lyme disease (LD) which is the most prevalent tick-borne disease in Europe and North America [8, 9]. LD may cause severe symptoms with manifestations in the skin, joints, nervous system and heart tissue in humans as well as in companion animals, especially in dogs [45–48]. *Ixodes ricinus* is known to be the main vector in Europe, whereas *I. scapularis* is the main vector in North America, and *I. persulcatus* in Eurasia [49–51]. The prevalences of *B. burgdorferi* (*s.l*.) in *I. ricinus* in Europe differ regionally. Studies from Europe, e.g. France [52, 53], the Netherlands [54], Slovakia [55] and Austria [56], show infection levels in *I. ricinus* ticks ranging from 3.3 to 22.5%. Earlier studies from Germany also showed high prevalence ranging from 11 to 36.2% in different regions of the country [57–59]. The present study confirms *I. ricinus* as the main vector for *B. burgdorferi* (*s.l*.), as the prevalence from this study was in line with previous studies from Europe [52–56]; however being lower than in previous studies from Germany (5.5%) [57–59]. The absence of *Borrelia burgdorferi* (*s.l*.) in questing *I. ricinus* larvae suggests a non-existent or insufficient transovarial transmission path [60]. However, transstadial transmission in ticks is verified [61]. Previous studies reported significantly higher prevalence for *B. burgdorferi* in adult *I. ricinus* ticks than in nymphs [52, 56, 59]. Our results are in contrast to these findings as *I. ricinus* nymphs were significantly more frequently infected than *I. ricinus* adults. Although in the past, spirochetes were detected in 11% of adult *D. reticulatus* ticks by immunofluorescence microscopy employing an antibody against *B. burgdorferi* [62], this non-specific method may likewise detect similar spirochetes such as *B. miyamotoi* [63]. Moreover, other study confirmed that *D. reticulatus* is not a suitable vector for *B. burgdorferi* (*s.l*.) [64, 65]. In our study, none of the *D. reticulatus* ticks examined tested positive for *B. burgdorferi* (*s.l*.); this supports the view that *D. reticulatus* is of minor importance in the natural life-cycle of this pathogen complex.

More than 40 vertebrate species, in particular birds and small mammals like rodents, are considered as reservoir hosts for *B. burgdorferi* (*s.l*.) in Europe [12, 13]. Previous studies from France, Ireland and Austria showed prevalence of *B. burgdorferi* (*s.l*.) in small mammal species ranging from 2.3 to 24% [66–68]. The infection level in small mammals in the current study was slightly higher than these obtained in earlier European studies (31.3%). In present research, each species belonging to the order Rodentia was positive and with high prevalence of *B. burgdorferi* (*s.l*.) (25.4–62.5%), whereas the insectivores (1 *Talpa europaea* and 5 *Sorex araneus*) and the carnivores (1 *Mustela nivalis*) were all negative. These findings are in line with a study from Austria where all rodent species were positive for *B. burgdorferi* (*s.l*.) and also with high prevalence (13.3–77.0%) [68]. The prevalence of spirochetes in rodents from this study was high and independent from their tick burden, and moreover significantly higher than in questing *I. ricinus*. These results therefore support the hypothesis that the rodent species studied are potential reservoirs for *B. burgdorferi* (*s.l*.). They are known to harbour *B. japonica*, *B. afzelii*, *B. bissettii* and the NT29 ribotype as well as the OspA serotype A of *B. garinii* [69].

*Borrelia afzelii* was found in all five small mammal samples. Studies from other European countries confirm that *B. afzelii* is a genospecies which is associated with rodents [70, 71]. In Europe, MLST was performed for the identification and genotyping of *Borrelia* spp. in rodents from central Slovenia [72], questing *I. ricinus* ticks from Norway [73] and the UK [74], and ticks and rodents from France [75, 76]. In Germany, the MLST method has thus far been used in research on phylogenetic relationships and global evolution of the *B. burgdorferi* (*s.l*.) species complex [77], and on the population structure and pathogenicity of *B. afzelii* and *B. burgdorferi* (*s.s*.) [78]. To our knowledge, this is the first study using MLST for the detection of allelic combinations of *B. burgdorferi* in small mammals from Germany. The analysis of the eight housekeeping genes, i.e. *nifS*, *uvrA*, *clpA*, *clpX*, *rplB*, *recG*, *pyrG* and *pepX*, revealed ST 165 and 559, both sequence types belonging to *B. afzelii*. These sequence types were described earlier in *I. ricinus* ticks from Latvia, Slovenia and France according to *Borrelia* spp. MLST database (http://pubmlst.org/bigsdb?db=pubmlst_borrelia_isolates&page=profiles).

Rickettsiae of the spotted fever group may cause a variety of clinical symptoms such as lymphadenopathia, fever and headache in humans [79]. In Europe, there are several different species of varying pathogenic potential (*R. aeschlimannii*, *R. conorii*, *R. helvetica*, *R. massiliae*, *R. monacensis*, *R. raoultii*, *R. sibirica* and *R. slovaca*) [15]. In the present study, *Rickettsia* spp. were detected in all collected tick species (*I. ricinus*, *I. trianguliceps* and *D. reticulatus*). Results from France, the Netherlands, Austria and Poland showed infection levels in *I. ricinus* ticks ranging from 1.4 to 41% [80–83]. The prevalence obtained in the present study is in line with these findings. High infection rates (11–50%) for *Rickettsia* spp. in *D. reticulatus* were detected in previous investigations from the UK, Slovakia and Croatia [84–86]. The infection level in the present research is higher (70.5%), though not as high as in a previous study by our group (85.6%) which was conducted in the same study sites [27]. Transovarial and transstadial transmission of *Rickettsia* spp. have been described in ticks. Moreover, horizontal transmission during feeding on a bacteriaemic host and co-feeding of *Rickettsia*-positive arthropods were also demonstrated [87, 88]. *Dermacentor reticulatus* is known to be the main vector of *R. raoultii*. As the prevalence in adult *D. reticulatus* ticks was very high but much lower in small mammals, it is probable that transovarial transmission is the main transmission path in *D. reticulatus* and that rodents are not of primary importance for the maintenance the natural circulation of *R. raoultii*.

The prevalence of *Rickettsia* spp. was significantly higher in *D. reticulatus* than in *I. ricinus* and in small mammals, pointing out that *D. reticulatus*-related rickettsiae are maintained independently from a vertebrate reservoir in nature, in contrast to *I. ricinus*-related rickettsiae. In Europe, there are very few studies about the maintaining and distribution of *Rickettsia* spp. in wild small mammals [32, 33, 81]. In Germany, two studies revealed the occurrence of *R. helvetica* in *A. agrarius*, *A. flavicollis* and *M. glareolus* [27, 32, 33]. In the present study, *Rickettsia* spp. was also found in these three rodent species. The study sites of the current research were earlier investigated for *Rickettsia* spp. by our group. These preliminary studies revealed high prevalences in *D. reticulatus* (56.7–85.6%), *I. ricinus* (13.4–17.5%), and small mammals (28.6%) [27, 38]. The prevalence rates for *Rickettsia* spp. in the present study are in line with earlier findings for *D. reticulatus* (70.5%), however slightly lower for *I. ricinus* (11.4%) and small mammals (25.3%). In previous investigations on small mammals from Germany, *R. felis, R. helvetica*, *R. monacensis* and *R. raoultii* were detected [27, 33]. Our results confirmed the occurrence of all mentioned *Rickettsia* spp. except for *R. monacensis*. All *R. raoultii*-positive rodents were infested with *D. reticulatus*, the main vector for *R. raoultii*. Interestingly, the *D. reticulatus* tick burden was positively correlated with the prevalence of *Rickettsia* spp. in *M. glareolus. Myodes glareolus* had a seven times higher chance of encountering *Rickettsia* infection while being infested with *D. reticulatus* in comparison to *M. glareolus* without *D. reticulatus*. Comparisons of the prevalence of *Rickettsia* spp. in small mammals between the years 2012–2014, revealed significantly lower infection rates in 2013 than in 2012 and 2014. Interestingly, none of the small mammals captured in 2013 was infested with *D. reticulatus*. This leads to the assumption that small mammals infected with *D. reticulatus-*related rickettsiae are rather incidental than potential reservoir hosts.

## Conclusions

The prevalence for *B. burgdorferi* (*s.l*.) in small mammals was high (> 30%) and independent of tick abundance, suggesting small mammals as reservoirs. To our knowledge, this is the first detection of *Borrelia* spp. sequence types in small mammals from Germany, revealing ST 165 and ST 559 which belong to *Borrelia* genospecies *B. afzelii*. Small mammals may also serve as reservoirs for *I. ricinus*-transmitted *Rickettsia* spp. Bank voles (*Myodes glareolus*) had a seven times higher chance of encountering *Rickettsia* spp. infection while being infested with *D. reticulatu*s in comparison to *M. glareolus* without *D. reticulatus*. As the prevalence in questing adult *D. reticulatus* was very high (> 70%) but much lower in rodents (*c*.25%), a potential reservoir function of bank voles is unlikely. The prevalence of *R. raoultii* in *M. glareolus* can be a result of infestation with infected *D. reticulatus*. We suggest that transstadial (and likely transovarial) transmission in *D. reticulatus* is the main mode of maintenance of *R. raoultii* natural life-cycle.

## Additional files

Additional file 1: Batch of *Borrelia afzelii* sequences according to the genes: *clpA*, *clpX*, *uvrA*, *nifS*, *rplB*, *recG*, *pepX*, *pyrG* of sequence type 165. (DOCX 17 KB)
Additional file 2: Batch of *Borrelia afzelii* sequences according to the genes: *clpA*, *clpX*, *uvrA*, *nifS*, *rplB*, *recG*, *pepX*, *pyrG* of sequence type 559. (DOCX 16 KB)

## Abbreviations

CI: Confidence interval
MLST: Multi-locus sequence typing
OR: Odds ratio
qPCR: quantitative polymerase chain reaction
SFG: Spotted fever group.
