# Supplementary material for: The enzootic life-cycle of Borrelia burgdorferi (sensu lato) and tick-borne rickettsiae: an epidemiological study on wild-living small mammals and their ticks from Saxony, Germany
Source: Parasit Vectors. 2017 Mar 13;10:115. doi: 10.1186/s13071-017-2053-4 (PMC5346851; doi:10.1186/s13071-017-2053-4)
Supplement: Additional file 2: — Batch of Borrelia afzelii sequences according to the genes: clpA, clpX, uvrA, nifS, rplB, recG, pepX, pyrG of sequence type 559. (DOCX 16 KB) [file 13071_2017_2053_MOESM2_ESM.docx]

**Additional file 2**. Batch of *Borrelia afzelii* sequences according to the genes: *clpA, clpX, uvrA, nifS, rplB, recG., pepX, pyrG* of sequence type 559

clpA

>clpA_36

AAAAAAGAATTCATTATACATGATAGTTTAGTATTTGATTTGATATTAAATATAAAATTATTAAAATTCAATTTACTTGCCAATAGAAGTACTATTGGCATATTTGCTTTTATTGGTGCTTCTGGAGCGGAAAAATGCAAATTGACGGATATTTTATCAGAAGAGTTTAAAATTCCGAAATTTAATCTTAATATGGGCGAGTATAGTGATTTTACTTCTCTTGATAGATTGATTGGCCCTGTTTTAAGTAATGATGGGTATTATGAATCTACTAGATTTTTCAAATTTTTAAACAAGTCTTCTAATTCTATTATTTTCCTATCAGATTTTGATAAATGTAATAAAAGGGTTTTAGATTTTTTTTTAGAGGGGTTTAGAACTGGCAAGCTTTTTGATGGTCTTGGGAAAAAGGTGAGCTTGTCAGAAAGTTTAATAGTAATAAGTGTCAATGCTGAGAGTAATGAGCTTAATAGTATTGGCTTTAAAAATAAAATGGCAGGGGAAAATGATTTTGATCTTATCTTAGAGAAGAGATTTCCCAATGAATTTTTAGAGTTAATAGATTATGTGTTTGTATTT

clpX

>clpX_24

GCTGGATATGTTGGTGAGGATGTAGAAAATATTTTACTTAAATTGATACATGCTGCTCATGGGGATGTTAGTCTAGCTGAGAAAGGGATTATTTATATAGATGAAATAGATAAAATTGCTAAAAAAAATGAAAACGTTTCAATAACAAGAGATGTGTCTGGAGAAGGGGTTCAACAAGCTTTGTTAAAGATAATCGAGGGTACTATTGCTAATGTTCCCCCAAGAGGTGGTAGGAAACATCCTTATGAGGATACTATTGAAATTAATACTCAAAATATACTCTTTATATGTGGTGGTGCTTTTGTTGGGCTTGAAAATATTGTTAAGAATCGAATAAATAAAAGTTCTATTGGGTTTTCAGCAATTGAAAAAAAGAATATAAGAGATGACACTTCATTAAAGTATTTGGAAATGGAAGATTTGATTAAATTTGGTTTAATACCAGAGTTTGTTGGTAGACTTCCTGTACATTCGTATCTTGAAAAGCTAAGTAAAGAAGATTTGATTAAAATATTAGTTGATCCTCAAAATTCTATTGTTAAGCAGTATTATCATATGTTTAAAATGGACAATGTTGAATTAGTCTTTGAAAAAGATGCTTTAGAATCAATTGTAGATGAGGCT

uvrA

>uvrA_28

TCAGGTAGTTTATCCGGGGGTGAGGCTCAGCGTATTAGGCTTGCTACTCAAATAGGGTCAGCACTTTCGGGTGTTATTTATGTTCTTGATGAGCCAAGCATTGGCCTTCATCAAAGAGATAATGAAAAATTAATCTCTACTCTTGTTAATCTTAAAAATCTTGGCAATACGGTAATTGTTGTTGAGCATGATGAGCAAACTTTGCGTACCGCAGATTATATTATTGATATGGGCCCTGGTGCTGGAATTCTTGGAGGGGAAATAGTTGCAAAGGGAACCTTAATAGATATTTTAAATAGTAAAAATAGTTTAACTGGTCAATATTTGAGTGGTGAGTTTAAAATAGATGTTCCAAGTTCTAGAAGAAAGACAGATAAGGGAGAGATTTTGCTTTTAGGTTCTAATAAAAACAATCTTAAAAATATAGACGTAAGTATTCCTTTGGGAGTTTTCACTGTAATAACAGGTGTTTCTGGTAGTGGAAAAAGTACTTTGCTTAACGAAGTGTTATATCCAGCTCTTGACAGCAGATTAAAGCTTGATAGAAAGTATTGTGATGGCTTTAAAGAC

nifS

>nifS_23

TTTGACAATAAGCATATAATTTATTTTGACAATGCAGCAACATCTCAAAAACCCAAAAAAGTAATTTATTCAAGCATTGAATATTATGAAAATTATAACGCAAATGTACACAGAAGCGGTCACAAATTTGCAATTCAATCTAGCATAAAAATAGAAAAAACAAGAGAACTTGTGAAAAATTTCATTAATGCAGAATCTGCAAAAAATATAATATTTACCTCTGGAACTACAGATGGAATTAATTCTGTTGCAAACTCATTTTTTTACTCAAAATACTTTAAAAAAAAAGATGAAATTATTCTTACAACTCTCGAACATAATAGTAATTTGCTCCCGTGGGCAAATCTTGCAAATTTAGCTAATCTAACAATTAAATTCGCTAAATTTAATGAAATGGGAATTATTACTCCTGAAGAAATTGAAAAACTTATTACAGAAAAAACAAAGCTCATCAGTATTTCAGGAATAAATAATACCTTGGGAACCATTAATGATCTAGAATCTATTGGAAAAATCGCAAAAAAATACAATATAAGTCTTTTTGTAGATGCTGCGCAAATGGCA

rplB

>rplB_22

GGTAATGATCCTTTGAAATCTTTAACAAAAGGTAAAAAATTTAAATCGGGCAGAGATTCTTCTGGTAGGATTAGTATTAGAAGAAGAGGTGGTGGGCATAAGAGAAAGTATAGGTTGATTGATTTTAATCGAAGAGATAAATTTAGCATTCCCGCTCGAGTTGCTTCTATTGAATATGATCCTAATAGAAGTGCTAATATAGCTTTGCTTGTTTATAAAGATGGAGAAAAAAGGTATATTATTTCTCCTAAAGACATTAAAGTTGGAGATGTTTTGGAAAGTGGTCCAAATGCCCCAATTAAAATTGGTAATGCCTTGCCCCTTGAAAACATTCCTATTGGAAGAACTGTTCACAATATCGAACTTAATGTAGGAAAGGGTGGGCAGCTTGTAAGAAGTGCTGGGGGGTATGCTATGATACTTGCTTCTGATGGGAATTATGTTACTGTAAAATTGTCATCTGGTGAGGTGAGGTTAATTTTCAAAAAATGCATTGCAACAATTGGTGAAATTGGAAATGAAGATTATGTCAATGTTTCTATAGGAAAAGCTGGTAAAAGTAGGTGGCTTGGTAGAAGACCTAAGGTTAGAGGTGTTGCCATGAATCCTGTTGACCATCCGCAT

recG

>recG_27

GCTCGTCAACATTATGATAATTTATCCAACATATTGTCCTCTTTTAACATTTCAGTGACTCTTTTGACTGGTAGTTTGAAAAAGAGGGATAAGGAGCAAGCGTTAGAAAGTATTAAAAGCGGAGCTTCTGGTTTAATAGTTGGAACACATGCTATTTTTTATGAAAGCACAGAATTTAAAAGATTAGCATATGTTATTATTGACGAGCAGCATAAATTTGGAGTTGTTCAAAGGGAAGAGCTTAAAAACAAAGGAGAAGGGGTAGATATGCTTTTAATGTCTGCAACACCTATTCCTAGAAGCTTTGCGTTAACTCTTTTTGGGGATCTTGAAATTTCATTTATTAAGACCTTACCTAAGGGGCGTTTACCTATTACTACTTATTTAGCAAAGCATGGCAATGAAGATAAAGTTTATGAGTTTTTAAGAAAAGAACTTGCAAAGGGTCATCAGGTTTATTTTGTTTATCCATTAATTTCATCTTCGGAAAAATTTGAATTAAAAGACGTTAATAATATGTGTTTAAAATTAAAAGAAGTGTTTGGCGAATATGTTGTTGACATGCTTCATTCTAAGTTGCCATCTGATTTGAAAGAAGAAATTATGAAAAATTTTTATTCTAAAAAAGTAGATATTTTGGTGGCTACTAGT

pepX

>pepX_86

TATAATACTCATGACAATTTAACAGTAATCAACAGCACTAAAAAAACCATAAAAGAAAATATCTTAGAACAACTTGGAATAAAATATGAAAATTTTCTATCTTGTGATTTAATATTCACAGAATCACAACCCTCAAAAATAATAGGAACTGAAGGAGAATTTTTAGCTTCTAAAAATCTTGATAATAAATCGGGATGCCATGCAATCATGAACTCTTATGTTCATACAAGTAATAATAAAAATAAAATAGCTGTATTTTTTGATAACGAAGAAGTAGGATCTTTAACATCAAGGGGCGCTGATTCCAATTTTTTATCAGAAGTTTTAGAAAGAATCGATCTTGCCCTTAACTTAACCAGAGAAGAGCATTTAATAAAAACAAACAAATCATTTAATATATCGATTGACAGCGTTCACGGAATTCATCCAGGATATGCATTCAAACATGATCCAAACTATCAAGCGACTCTAAGTAAAGGTGTAGTTGTAAAAAATAGCGCCAATTTTAGATATGCAACAACTTCAACGGGATTTGCAAAATTAAAAAACTTAGCTATTGAAAATAATATT

pyrG

>pyrG_20

GGAAGTGGTAATATTGCTTTTATTCATTTAACCTATGTACCAAGTCCAGTTGGAATTAATGAGCAAAAATCTAAACCCACTCAACAAAGTGTTAAAACTTTAAATAAAGCAGGCATTTTCCCTGATTTGATTATTGCTAGAAGTTCTCAAGTATTGACAGATCAAATCAGAAAAAAAATAGCAATGTTTTGTAATGTTGAGAGTACTTCTATTATTGATAATATTGATGTTTCTACTATTTATGAAATTCCCATATCTTTTTACAAACAAGGTGTGCATGAGATTTTAAGTTCTAAGTTGAATATTAAGGTTGACCCAAAAATAGAAGAGCTTTCAAGGCTTGTAGGGATTATAAAATCTAATTTTTTTGTGCCTAAAAAAATTATTAATATTGCTATTTGTGGTAAGTATGCTGAACTTGATGATTCTTATGCGTCAATTAGAGAGTCTTTGGTTCATGTTGCAGCTAATTTGGATTTACTTATTAAAAGCACTCTCATTGATTCTAATGATTTAAATGAAAGCTGTTTGAAGGATTTTGACGGTATTATTGTTCCCGGCGGCTTTGGAGGTAAAGGATATGAGGGCAAGATTATTGCTATT
